# Design of Convolutional Neural Network Processor Based on FPGA Resource Multiplexing Architecture

**DOI:** 10.3390/s22165967

**Published:** 2022-08-10

**Authors:** Fei Yan, Zhuangzhuang Zhang, Yinping Liu, Jia Liu

**Affiliations:** 1 School of Automation, Nanjing University of Information Science and Technology, Nanjing 210044, China; 2 Jiangsu Collaborative Innovation Center for Atmospheric Environment and Equipment Technology, Nanjing 210044, China; 3 School of Atmospheric Physics, Nanjing University of Information Science and Technology, Nanjing 210044, China

**Keywords:** FPGA, resource reuse, parallel processing, handwritten digit recognition

## Abstract

As CNNs are widely used in fields such as image classification and target detection, the total number of parameters and computation of the models is gradually increasing. In addition, the requirements on hardware resources and power consumption for deploying CNNs are becoming higher and higher, leading to CNN models being restricted to certain specific platforms for miniaturization and practicality. Therefore, this paper proposes a convolutional-neural-network-processor design with an FPGA-based resource-multiplexing architecture, aiming to reduce the consumption of hardware resources and power consumption of CNNs. First, this paper takes a handwritten-digit-recognition CNN as an example of a CNN design based on a resource-multiplexing architecture, and the prediction accuracy of the CNN can reach 97.3 percent by training and testing with Mnist dataset. Then, the CNN is deployed on FPGA using the hardware description language Verilog, and the design is optimized by resource multiplexing and parallel processing. Finally, the total power consumption of the system is 1.03 W and the power consumption of the CNN module is 0.03 W under the premise of guaranteeing the prediction accuracy, and the prediction of a picture is about 68,139 clock cycles, which is 340.7 us under a 200 MHz clock. The experimental results have obvious advantages in terms of resources and power consumption compared with those reported in related articles in recent years, and the design proposed in this paper.

## 1. Introduction

In recent years, convolutional neural networks (CNNs) have become a research hotspot in the fields of image classification, target detection, and speech recognition [1,2]. The increasing amount of data and computation of CNN models leads to the fact that CNN models can only be used on some specific platforms, but not on low-cost and portable small hardware. Therefore, lightweight neural network architectures and reducing the hardware resources and power consumption of deployed hardware platforms have become issues of concern.

In 2016, SqueezeNet used a convolutional dimensionality reduction method, which enabled the model to be reduced to 4.8 MB while the accuracy of image classification was 80.3 percent [3]. MobileNet, proposed by Google in 2017, had a model parametric number of 4.2 MB and the accuracy of image classification was 73.7 percent [4,5]. Subsequently, network structures such as ShuffleNet and ESPNet reduced the number of model parameters and improved the accuracy of image classification through techniques such as convolutional dimensionality reduction, channel separation, and conversion of depth to width [6,7]. The gradual development of lightweight network models has reduced the difficulty of deployment on hardware to some extent; however, these are still some distance away from practical applications.

Currently, the more commonly used CNN hardware carriers are the central processing unit (CPU), graphic process unit (GPU), application-specific integrated circuit (ASIC), and field programmable gate array (FPGA), etc. [8,9]. Among them, CPUs and GPUs face the problems of high power consumption, high cost and inability to miniaturize; application-specific integrated circuits have the disadvantages of a long cycle time, high cost and poor flexibility; FPGAs, with their parallelism, low power consumption and reconfigurability, are one of the promising platforms for CNN deployment. Therefore, many researchers have proposed the optimization of the structure and scheme of CNN algorithms based on FPGAs. Chen et al. cached the intermediate data to internal storage in order to improve parallelism, but it was not flexible enough and has poor generality [10]; Lu et al. proposed a reduction in the application of the digital signal processor (DSP) and proposed a Winograd algorithm for computing, which, to some extent, reduces the amount of computation, but is limited [11,12,13,14]; Zhang et al. proposed fast Fourier transform (FFT) to improve computational performance, but only for larger convolutional-kernel computation [15].

In this paper, we propose a convolutional-neural-network-processor design based on an FPGA-based resource-multiplexing architecture, taking the classic handwritten digit recognition CNN as an example. The main contribution of this work is divided into two parts: one part is the preprocessing of the CNN architecture for hardware deployment implementation, which, in turn, completes the hardware implementation of the processor with resource reusability architecture; the other part is the hardware deployment of the CNN layer structure on FPGA, which performs layer fusion and parallel processing for the resource-reusability architecture to reduce the computational- and hardware-resource overhead. Finally, the resources and power consumption used by the CNN processor of this paper’s architecture are compared with related articles of the same type. The main FPGA resources used greatly reduce the consumption of resources such as LUT, DSP and BRAM compared with the processor design of Tong et al. [16,17,18,19,20]. In terms of power consumption, the power consumption of the processor in this paper is much lower than the power consumption of the processor designed by Tong et al. Therefore, the processor designed in this paper has more obvious optimization in terms of hardware resources and power consumption.

## 2. Architecture Design of CNN

The basic structural unit of a CNN includes a convolutional layer, pooling layer, activation layer and fully connected layer. The main function of the convolution layer is to extract the features in the image information and output the feature map by convolving the image data in the sliding window with the weight parameters in the convolution kernel; the main function of the pooling layer is to simplify the feature map and reduce the model parameters by replacing the data in the feature map with the result value after the function operation using the pooling function; the main function of the fully connected layer is to connect with the output of the previous layer; and the main function of the fully connected layer is to connect with the features output from the previous layer and perform a nonlinear combination of the features extracted from the previous layer, so as to achieve the learning goal.

Since the inference phase of the CNN needs to be deployed in the FPGA, the CNN architecture is designed to meet the accuracy of CNN prediction while keeping the amount of data and computation as small as possible. The CNN design of this system has the following features:

1. Minimized number of filters per convolutional layer and fully connected layers without degrading classification performance, and use of small convolutional kernels of 3×3;
2. Bias parameters are not used. Since the parameters need to be converted from floating point to fixed point into the FPGA for processing, the bias parameters are not used to avoid the accumulation of errors in the fixed-point parameterization, thus reducing the impact on recognition accuracy;
3. Using maximum pooling, GlobalAvgPooling is replaced with GlobalMaxPooling; it is easier to implement maximum pooling operations in hardware compared to average pooling;
4. Use the simple activation function ReLU, as other activations, including division, power and other functions, are difficult to implement in hardware;
5. Minimized number of heterogeneous layers to allow FPGAs to implement resource-reuse and parallel-processing techniques for large amounts of data.

**Figure 1:**
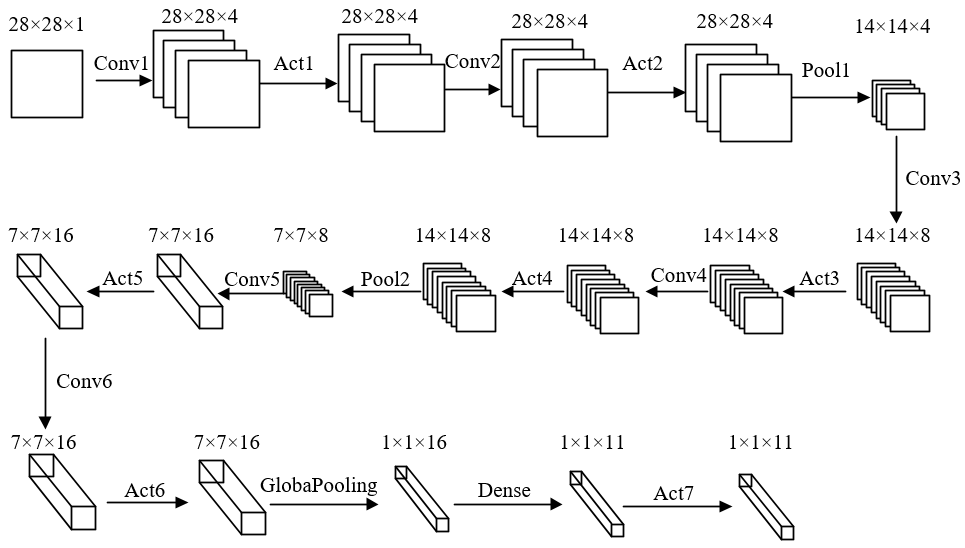
Convolutional neural network model.

The CNN of this system is built using Python based on Tensorflow and Keras frameworks, mainly consisting of six convolutional layers, seven activation layers, three pooling layers and one fully connected layer. The CNN removes a large number of fully connected layers and bias terms, and the convolutional layers use all 3×3 convolutional kernels. The total number of parameters of the optimized CNN is reduced from 25,000 to about 4676, and the accuracy of the convolutional neural network reaches 97.3 percent on the MNIST dataset, as shown in Figure 1.

Among them, the total number of inputs, outputs, convolutional kernel sizes and parameters for each layer of the CNN model is shown in Table 1. To maximize the resource-multiplexing architecture of the FPGA hardware resources, the convolutional kernel sizes of the convolutional layers are all 3×3, and the fully connected layer uses a small convolutional kernel of 1×1.

## 3. Hardware-Accelerated Design of CNN on FPGA

### 3.1. Overall System Architecture

The hardware part mainly consists of a Xilinx’s A7 series XC7A35T chip as the main control chip, DDR3 chip as the cache buffer unit, OV5642 camera as the video image real-time acquisition module, and LCD display as the graph display module and prediction-result display module.

**Figure 2:**
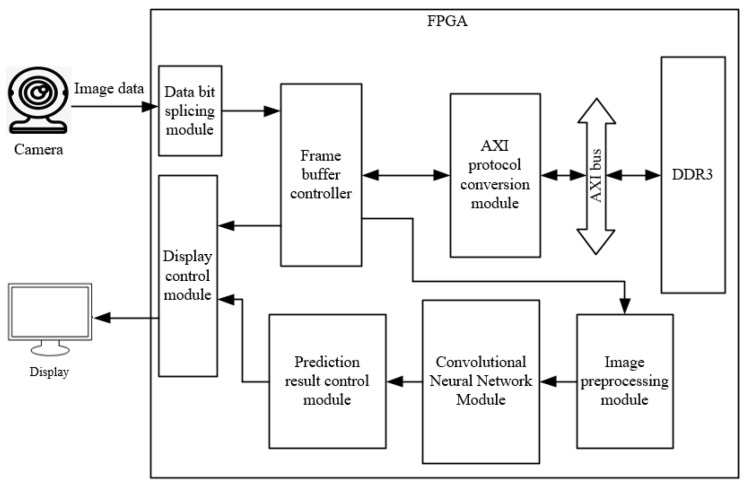
Overall block diagram of the system.

The overall block diagram of the system is shown as follows in Figure 2: first, the camera acquires image data, and the FPGA stitches the input image data into 16bit and outputs it to the frame cache controller; then, the image data is stored in the two addresses of DDR3 sequentially using a ping-pong operation, and the frame cache controller reads out the image data from DDR3 through AXI bus; finally, one way of the image data is displayed on the monitor in real time; the other way of the image data is sent to the image pre-processing module, and the processed 28×28 image is input to the convolutional-neural-network module for predictive classification, and the successful recognition result is displayed on the right side of the LCD in real time.

### 3.2. Data Caching and Interaction

**Figure 3:**
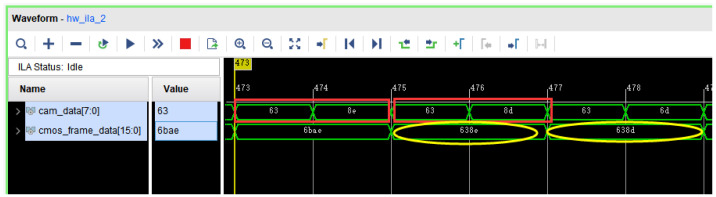
Comparison of results before and after bit splicing.

The OV5642 camera is used as a real-time video-image acquisition module with a resolution of 480×272. The image data input from the camera is cached in the FIFO of the frame cache control module after passing through the data bit stitching module, and is burst transferred to DDR3 via AXI bus, where the simulation diagram before and after bit stitching is shown in Figure 3.

Using dynamic address switching and a ping-pong operation to achieve the real-time smooth display of video images, even for high frame rates and large resolution images, can achieve real-time display effects, to avoid screen tearing, a broken phenomenon. Video images are displayed in real time on the LCD display.

**Figure 4:**
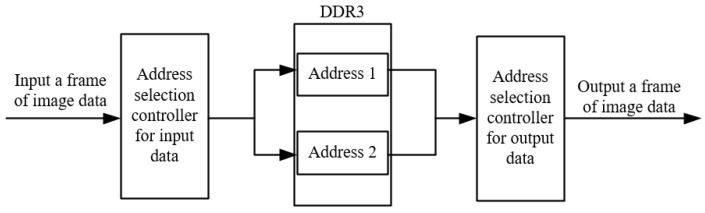
Schematic diagram of ping-pong operation of DDR addresses.

The ping-pong operation is carried out by setting two storage-space addresses in the DDR for storing two frames of images. They are used to store the input image data and the output image data, respectively. While the first memory space stores the input image data, the second memory space is used to output the image data. If both read and write data are completed, the read and write storage spaces are swapped. The main reason for using this ping-pong operation is that if only one storage space is used to input and output image data, the current frame of the image has not been read and output, and the next frame of the image data is input. The ping-pong operation of the DDR address is shown in Figure 4.

**Figure 5:**
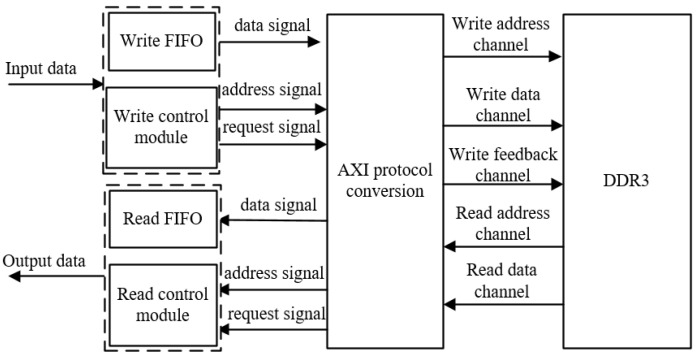
General block diagram of image-data caching and transmission.

Given that AXI is a burst data-transfer method, the write transfer of AXI in this paper is triggered by the amount of data cached in the FIFO. When the data stored in the FIFO is larger than the preset value, a burst transfer is performed to transfer the data cached in the FIFO to the DDR via AXI burst. Since the interface method of FIFO in this paper is Native, while the interface method of DDR3 is AXI4, a protocol conversion is required. Similarly, when reading image data from the DDR to send to the display for display, the data is read out from the DDR via AXI bus. Using the amount of data triggered by the FIFO cache, when the data stored in the FIFO is less than the preset value, the data in the DDR is cached into the FIFO via AXI burst transfer and then sent to the display control module for display. The overall block diagram of image data caching and transmission is shown in Figure 5.

### 3.3. Image Pre-Processing

**Figure 6:**
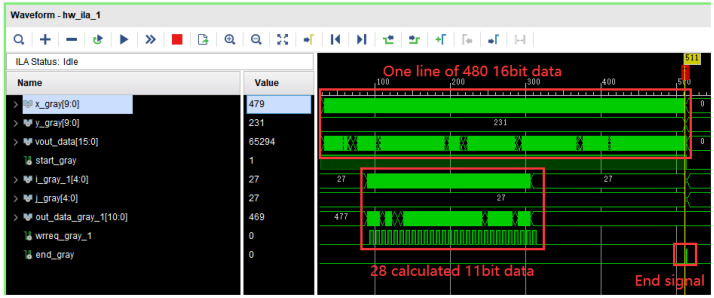
Simulation diagram of image pre-processing.

The image pre-processing module grayscales and image crops each frame that is read out in DDR3. Due to the specificity of human visual perception, a weighted average is used instead of a simple average. To facilitate the conversion at the hardware level, the following equation is used:

(1) BW=(8×G+5×R+3×B)/16

where *R*, *G*, *B* and *BW* represent the red channel, green channel, blue channel and grayed-out pixel values, respectively. Next, the grayscale map is trimmed. Since the MNIST image size is 28×28 pixels and the camera image resolution is 480×272, this scheme first crops the center part of the 480×272 image to 224×224 pixels; then, the image with a resolution of 224×224 is converted to a resolution of 28×28. The image with a resolution of 224×224 is first divided into 8×8 blocks, and then the average value of each block is calculated to form the corresponding pixel in the 28×28 image. Finally, the 28×28 image is input to the convolutional neural network module for classification, and the prediction results are displayed in real time on the right side of the LCD. The simulation diagram of the image pre-processing is shown in Figure 6.

### 3.4. Hardware Design of CNN Module

#### 3.4.1. The Overall Structure Design of CNN

**Figure 7:**
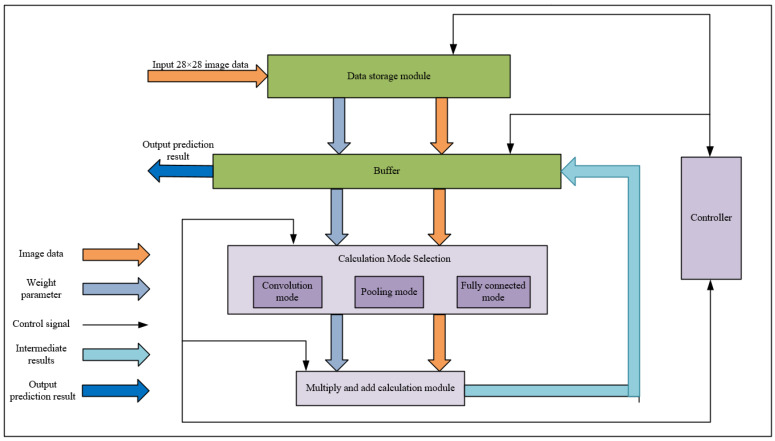
The overall hardware design structure of CNN.

The overall hardware design structure of CNN is shown in Figure 7, which mainly includes five parts: data storage module, buffer, calculation mode selection, calculation module and controller.The main function of the data storage module is to store the weight parameters and image data; the main function of the buffer is to cache the intermediate results of the calculation process; the calculation mode selection mainly includes convolution mode, pooling mode and fully connected mode, all three modes serve to prepare the intermediate result data and weight parameters required by the corresponding mode for the next step of calculation; the calculation module is mainly responsible for multiplying and adding the input image data and weight data; the main function of the controller is to control the order of each layer of calculation and the direction of data flow.

The workflow of the CNN module is, roughly, as follows:

1. The CNN module is initialized and the weight parameters are then stored to the data storage module, while the module waits for the 28×28 size image data to enter;
2. When the image data is input, the controller reads the image data and weight parameters into a buffer, which packs the data into a calculated format and sends it to the next level of calculation mode selection;
3. Through the control signal from the controller, the calculation mode selects the convolution mode to be performed, and the convolution mode determines how many convolution steps have been performed, so that the image data in the buffer is read out through the control signal, and the weight parameters are sent to the calculation module together with the image data;
4. The calculation module performs nine multiplication and eight addition operations on the image data and weight parameters, and the intermediate results of the calculation are output to the buffer until the end of the calculation in this layer;
5. After receiving the data of intermediate results, the buffer caches it in BRAM and waits for the controller to issue the next layer of calculation instructions;
6. When the next layer of calculation instructions is received, the buffer then sends the intermediate results and weight parameters to the calculation mode selection, and after the corresponding mode is selected, the data is sent to the multiplication and addition calculation module for calculation, and again, the results of the calculation are output to the buffer, and so on, until the calculation of all layers is completed; the buffer will output the prediction results.

#### 3.4.2. Design of Convolutional Layers

**Figure 8:**
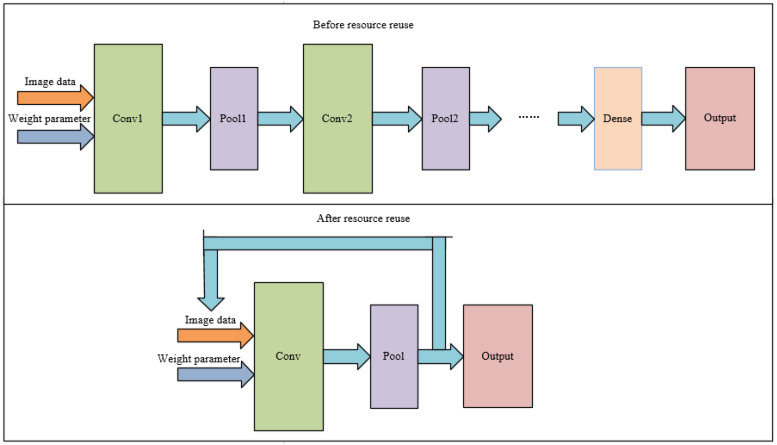
Structure diagram before and after resource multiplexing.

To maximize the resource reuse architecture, the convolutional layers designed in this system are all 3×3 convolutional kernels, which avoids the design of corresponding structures for convolutional kernels of different sizes and makes the designed computational modules universal. In view of the fact that the same layer architecture will occupy a lot of extra hardware resources, this system adopts a resource reuse architecture to reuse the same convolutional layer unit, which greatly reduces the consumption of hardware resources. The structure diagram before and after resource multiplexing is shown in Figure 8.

In the convolutional layer, the convolutional kernels in the neurons are all 3×3. The system is designed with a special convolutional computation module, which makes nine multiplications and eight additions in one clock cycle by blocking assignment, which greatly reduces its clock cycle compared to the traditional non-blocking assignment. The calculation formula for the convolution operation is:

(2) $$x_{j}^{n}=\sum_{\begin{array}{c} i=d \end{array}}y_{i}^{n-1}w_{ij}^{n}+b_{j}^{n}$$

(3) $$y_{j}^{n}=f\left(x_{j}^{n}\right)$$

In the formula, $y_{i}^{n-1}$ is the input feature map of the layer n−1, $w_{ij}^{n}$ is the convolution kernel of the layer *n*, $b_{j}^{n}$ is the bias value of the feature map of the layer *n*, *f*() is the ReLU activation function, and $y_{j}^{n}$ is the output of the *n*th layer feature map. Since the system design does not use the bias parameter, the parameter $b_{j}^{n}$ is not required.

In the hardware implementation, for the ZeroPadding layer, this system is implemented by the edge detection method, which reduces the clock cycles and the consumption of storage resources for this layer. For the activation layer, except for the last layer, which is a Softmax function, the ReLU activation function is used, which not only saves hardware resources, but also effectively removes negative points and is easy to implement in hardware. The formula of the ReLU activation function is as follows:

(4) $$f\left(x\right)=\left\{\begin{array}{c} x,x>0\\ 0,x\leq 0 \end{array}\;\right.\;$$

**Figure 9:**
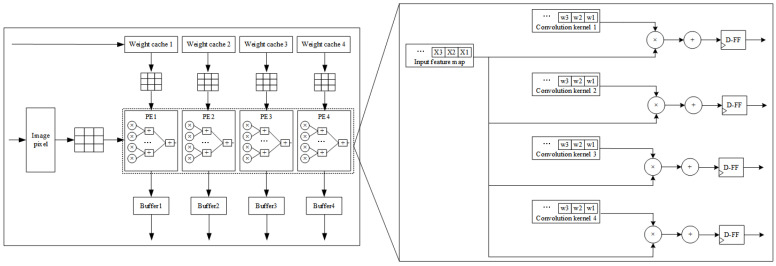
Parallel convolutional architecture design.

Meanwhile, this system is designed in hardware by the pipelining technique and parallel processing technique by analyzing the parallelism of the CNN convolutional layers. For example, in the second convolutional layer, the input has four images of 28×28 and 16 parameters of 3×3 convolutional kernel weights. If there is only one convolution block, the same set of image pixels needs to be multiplied and added four times with different 3×3 weight convolution kernels, which means at least four clock cycles are required. Therefore, this system deploys four such convolution blocks in parallel, which requires only one clock cycle to complete four multiplication and addition operations with the convolution kernel. The design of the parallel convolutional operation architecture is shown in Figure 9.

**Figure 10:**
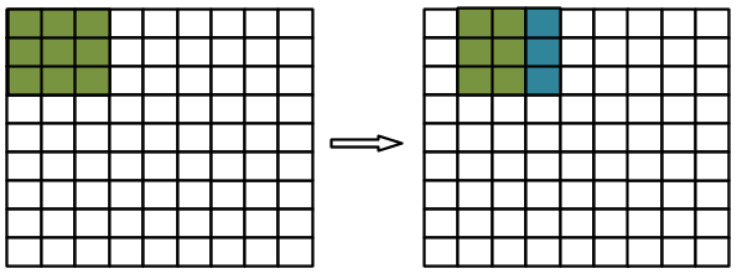
Shift operation of image data.

Since nine image pixel values are required to perform one convolution operation, this system design optimizes the calculation steps by using shift registers. For the nine neighboring pixel values of the previous operation step, only three pixel values need to be updated in the next step by keeping the six pixel values on the right side through shift registers. Therefore, only three new values are needed in each step instead of nine. The process is shown in Figure 10, where green is the image pixel value of the previous step and blue is the three image pixel values to be updated in the next step.

In addition, the edge of the image is detected according to the image timing. When the shift register moves to the right at the end of the row, the shift register is switched to the next row to read the image data, and then continues to move to the right. When the shift register reads to the end of the last row of the image, it means that the calculation of one frame of the image is completed, and the next frame is switched for calculation.

**Figure 11:**
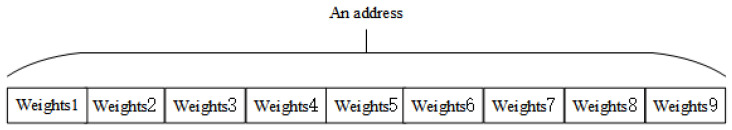
Storage of weight parameters.

For performing one convolutional operation with nine weight coefficients, this system is designed by storing the nine weight parameters in one address, as shown in Figure 11. When the convolution calculation process is performed, only one clock cycle is needed to extract all nine weight parameters, thus speeding up the calculation efficiency of the convolution or fully connected layer.

#### 3.4.3. Design of Pooling Layer

**Figure 12:**
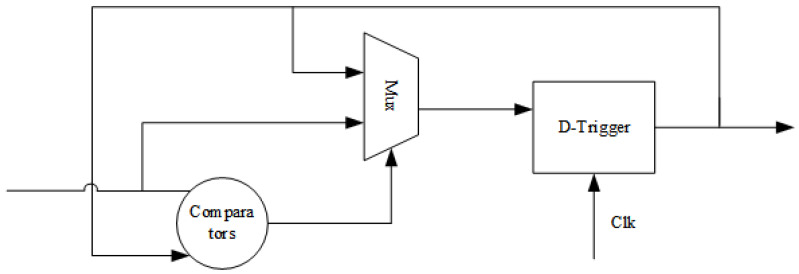
Hardware design architecture for maximum pooling.

This system design uses the maximum value pooling method for dimensionality reduction to reduce the computational effort. The pooling window is 2×2, and the original four values are replaced by the maximum of the four neighboring values. Compared with average pooling, maximum pooling is easier to implement in hardware. It also reduces resource consumption and does not produce a significant degradation in network performance. The maximum pooling unit mainly includes comparators, multiplexers and flip-flops, and its hardware design structure is shown in Figure 12.

#### 3.4.4. Design of Fully Connected Layer

The fully connected layer uses a 1×1 convolutional kernel with a total of 11 neurons, each containing 16 weights. The main operation of full connectivity is to extract the association between these features by a matrix vector product; the features extracted earlier, after nonlinear changes in Dense, and, finally, mapped on the output space. The calculation formula is as follows:

(5) $$y_{i}=\sum_{j=0}^{m}\left(W_{ij}\times x_{ij}\right)$$

**Figure 13:**
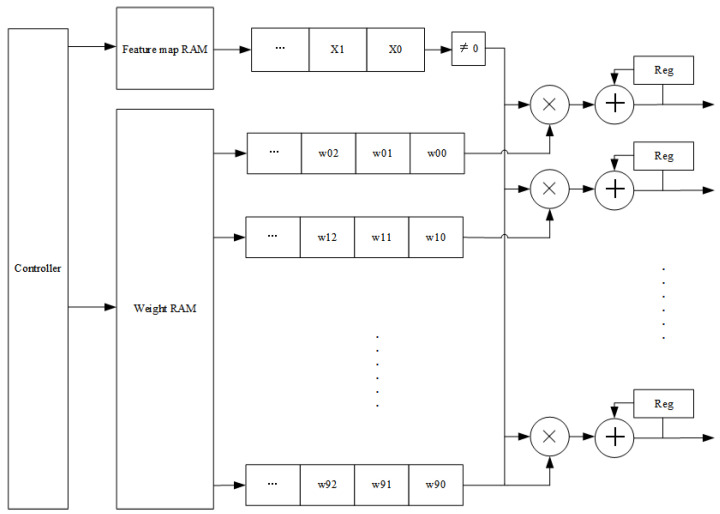
Hardware design architecture of the fully connected layer.

When the design is performed in hardware, the intermediate results and weights of the input are multiplied and accumulated, and then the classification results are output. Before performing the multiplication operation, we judge whether the intermediate result of the input is 0 or not, and then perform the multiplication operation. The computation module of the convolutional layer is reused, thus reducing resource consumption. Since the input of the computation module is nine weights and nine intermediate results, the 16 neurons are divided into two calculations, and eight multiplication accumulation calculations are performed once. The hardware design structure diagram is shown in Figure 13.

### 3.5. Optimization of the Design

**Figure 14:**
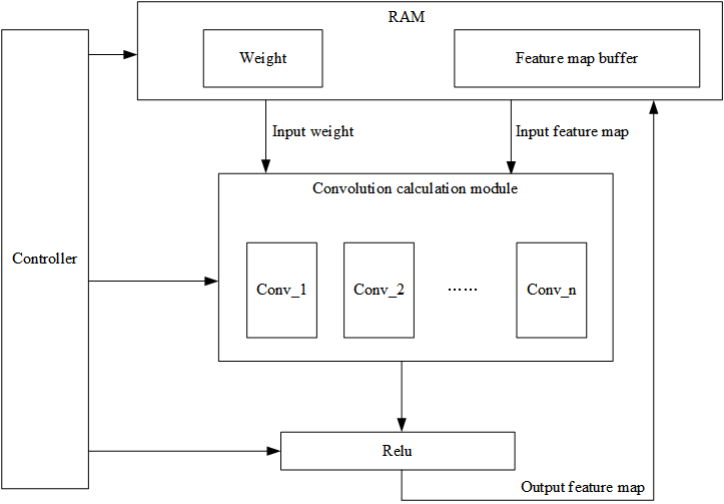
Convolutional parallel cyclic multiplexing structure.

Since the computation in CNN is mainly in the convolutional layer, a large amount of hardware resources are consumed in the hardware design process of the convolutional layer. Traditional CNN architectures often contain convolutional cores of different sizes, which require the design of different layer structures on FPGAs, thus increasing the resource consumption and power consumption of hardware [16]. The CNN architecture proposed in this paper has high regularity, and the convolutional layers all use small convolutional kernels of 3×3. Meanwhile, this paper proposes a convolutional parallel cyclic multiplexing structure in the hardware implementation process, as shown in Figure 14 below. The overall structure design of a CNN module on FPGA using a convolutional parallel cyclic multiplexing structure avoids the design of corresponding structures for different sizes of convolutional cores and the superposition of the same layer structure, which reduces the consumption of hardware resources and lowers the power consumption to a greater extent. The comparison of the resource consumption in FPGA between the traditional architecture and the handwritten digit recognition CNN of this paper’s architecture is shown in Table 2. Compared with the traditional CNN architecture, the LUT resources are reduced by about 82.7 percent, the BRAM resources are reduced by about 56.5 percent, and the DSP resources are reduced by about 98.9 percent.

**Figure 15:**
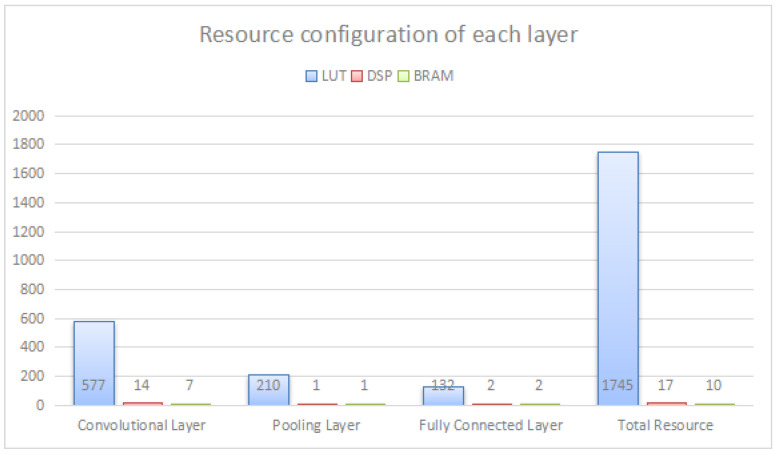
Resource configuration of each layer.

This design greatly reduces the consumption of hardware resources, thanks to the convolutional parallel cyclic reuse structure at the convolutional layer, and the resource allocation of each layer is shown in Figure 15. Among them, the LUT resources of the convolutional layer account for 33.1 percent of the total resources of the CNN module; the LUT resources of the pooling layer account for 12 percent of the total resources of the CNN module; and the LUT resources of the fully connected layer account for 7.6 percent of the total resources of the CNN module. It can be found that the hardware resources consumed by the convolutional layer of this design only account for a small portion of the total resources, and no longer account for a large portion of the total resources as in the previous designs [17].

## 4. System Experiment and Analysis

**Figure 16:**
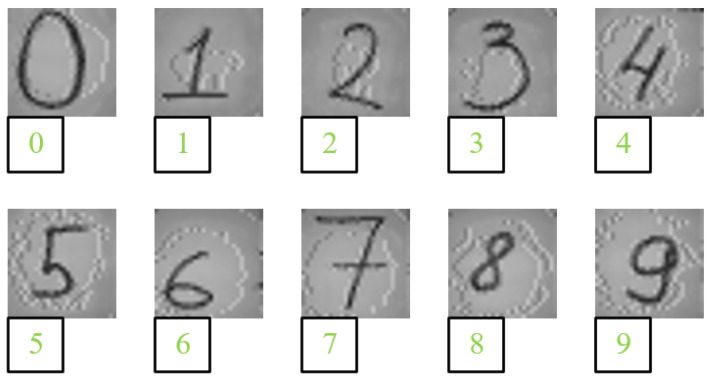
Test result chart.

After the design of the FPGA-based resource-multiplexing architecture neural-network processor is completed, in order to verify the network recognition effect of the processor, the recognized pictures are printed and the results printed below are the pictures after recognition. As shown in Figure 16, this design can realize the effective recognition of handwritten digits. In addition, the system can effectively recognize handwritten digits even when the image is rotated a little (within 30${}^{\circ }$) or interfered with by noise.

Since the handwritten numbers of different people will be different, the handwritten numbers from 0 to 9 of 10 students were collected for testing. After a test experiment of 100 handwritten digits, 97 handwritten digits were correctly identified, and the prediction accuracy is about 97 percent.

**Figure 17:**
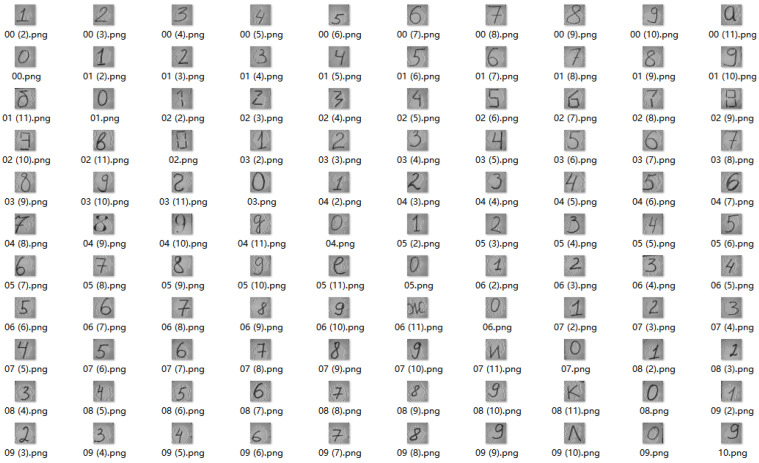
Experimental pictures of the test.

Considering that a camera is used to collect image information, the accuracy of the collected images may be reduced, which will affect the prediction accuracy. As shown in Figure 17, 10 pictures of handwritten digits 0 to 9 and 10 non-digital pictures were extracted from the dataset for testing experiments, with a total of 110 images. First, 110 images were converted into .txt format data in a batch; then, the .txt format image data were read into the simulation file for simulation testing; finally, the CNN module deployed on the FPGA could correctly identify 107 images, with a prediction accuracy of about 97.3 percent.

**Figure 18:**
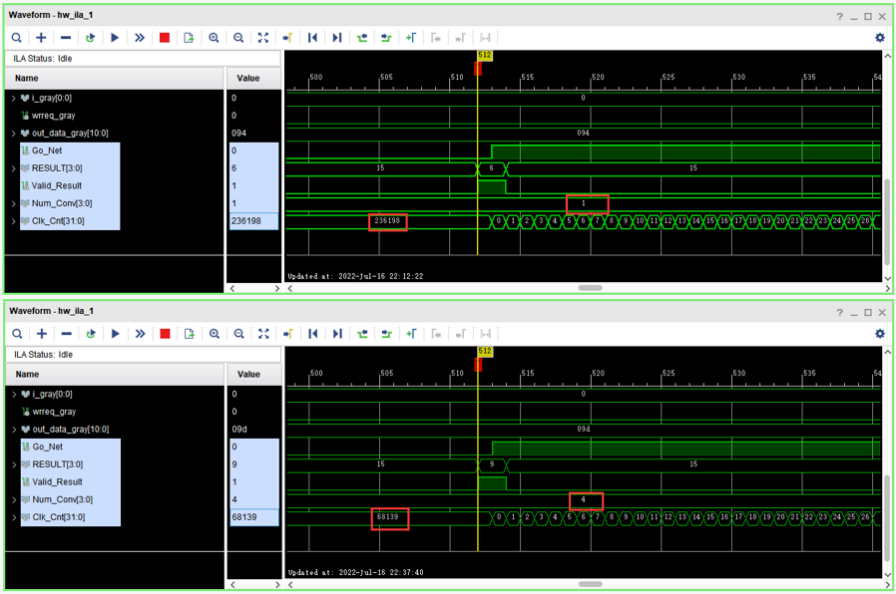
Predicted latency simulation diagram.

After deploying the CNN to the FPGA, the latency simulation diagram of predicting a picture is shown in Figure 18. When the signal line Go-Net is pulled high, the calculation starts; the signal line RESULT is the predicted result; Valid-Result is the result valid signal; the signal line Num-Conv is the number of parallel convolution blocks; the signal line Clk-Cnt is the number of delay clock cycles. When the number of parallel convolution blocks is 1, the prediction of a picture is about 236,198 clock cycles; when the number of parallel convolution blocks is increased to four, the prediction of a picture is about 68,139 clock cycles, and the speed is increased by 3.47 times. If the system clock is 200 MHz, the latency of predicting one image is about 340.7 us.

**Figure 19:**
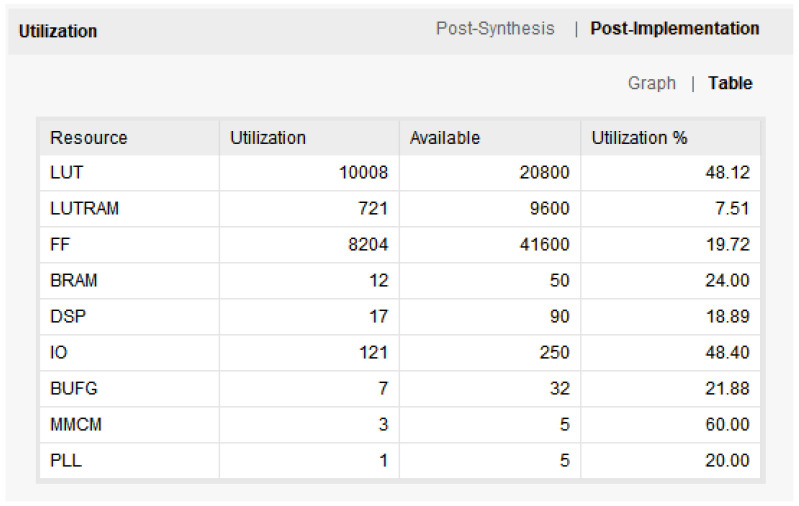
FPGA resource utilization.

**Figure 20:**
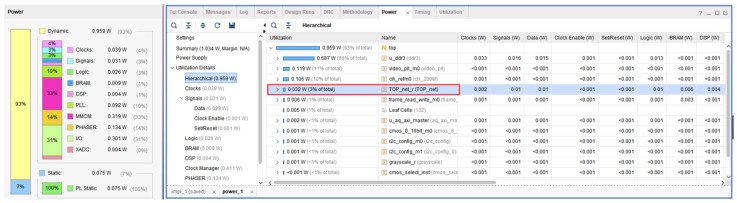
FPGA power consumption.

The total resource usage of FPGA in this system is shown in Figure 19. The FPGA power consumption of this system is shown in Figure 20. The left side of the figure shows the total power consumption of this system, and the right side of the figure shows the power consumption of the CNN module. The total power consumption of this system is about 1.03 W (dynamic 0.959 W + consumption 0.075 W), and the power consumption of the CNN module is about 0.03 W.

The resources and power consumption consumed by the CNN module design in this system are compared with other handwritten digit recognition literature in the following, as shown in Table 3 and Table 4. The resource consumption of this system is mainly LUT, DSP and BRAM, where 1745 LUTs are used, 17 DSPs are used and 10 BRAMs are used. The LUT resources of the model designed in this system are saved at least eight times more compared to the literature [18,19,20,21,22]; the DSP resources are saved at least three times more compared to the literature [18,19,20,21], and the resource consumption is similar compared to the literature [20]; the BRAM resources are all saved at least three times more compared to the literature [18,19,20,21,22]. The power consumption is reduced by at least 12 times or more compared to the literature [18,19,20,21,22]. There are more obvious optimizations in terms of resource-consumption and power-consumption design.

## 5. Conclusions

This paper proposes an FPGA-based resource multiplexing-architecture convolutional-neural-network-processor design, which aims to reduce the consumption of hardware resources and power consumption by CNN. In terms of the CNN architecture design, the CNN architecture proposed in this paper has high regularity, and the convolution layers all use 3×3 small convolution kernels; in the process of hardware implementation, this paper proposes a convolution parallel cyclic multiplexing structure. Through the design of these two aspects, the reasoning process of CNN is deployed on FPGA. It avoids the design of corresponding structures for convolution kernels of different sizes and the superposition of the same layer structure, which greatly reduces the consumption of hardware resources and power consumption.

The system takes handwritten digit recognition CNN as an example to design a convolutional neural network processor with a resource multiplexing architecture. Finally, the prediction accuracy of the processor is 97.3 percent; the consumption of hardware resources is significantly reduced; the total system power consumption is about 1.03 W, and the power consumption of the CNN module is about 0.03 W; it takes about 68,139 clock cycles to predict a picture, the delay time is about 340.7 us under a 200 MHz clock. Compared with the related articles reported in recent years, the experimental results have obvious improvements in hardware resources and power consumption.

## Figures and Tables

**Table 1:** Statistics of each parameter in the model.

	Input	Output	The Size of the Convolution Kernel	The Number of Convolution Kernels	Total Number of Parameters
Conv 1	28 × 28 × 1	28 × 28 × 4	3 × 3	1 × 4	36
Act 2	28 × 28 × 4	28 × 28 × 4	/	/	/
Conv 2	28 × 28 × 4	28 × 28 × 4	3 × 3	4 × 4	144
Act 2	28 × 28 × 4	28 × 28 × 4	/	/	/
Pool 1	28 × 28 × 4	14 × 14 × 4	/	/	/
Conv 3	14 × 14 × 4	14 × 14 × 8	3 × 3	4 × 8	288
Act 3	14 × 14 × 8	14 × 14 × 8	/	/	/
Conv 4	14 × 14 × 8	14 × 14 × 8	3 × 3	8 × 8	574
Act 4	14 × 14 × 8	14 × 14 × 8	/	/	/
Pool 2	14 × 14 × 8	7 × 7 × 8	/	/	/
Conv 5	7 × 7 × 8	7 × 7 × 16	3 × 3	8 × 16	1152
Act 5	7 × 7 × 16	7 × 7 × 16	/	/	/
Conv 6	7 × 7 × 16	7 × 7 × 16	3 × 3	16 × 16	2304
Act 6	7 × 7 × 16	7 × 7 × 16	/	/	/
Global pool	7 × 7 × 16	1 × 1 × 16	/	/	/
Dense	1 × 1 × 16	1 × 1 × 11	1 × 1	16 × 11	172
Act 7	1 × 1 × 11	1 × 1 × 11	/	/	/
Total	/	/	/	/	4676

**Table 2:** Comparison of resource consumption between traditional architecture and this paper’s architecture.

	Traditional Architecture [16]	This Paper’s Architecture
LUT	10,078	1745
BRAM	23	10
DSP	1485	17

**Table 3:** Comparison of FPGA Resource Utilization.

	This Article	Document [18]	Document [19]	Document [20]	Document [21]	Document [22]
Model	Resource Reuse Architecture	LeNet-5	RLeNet	LeNet-5	LeNet-5	RLeNet
Dataset	MNIST	MNIST	MNIST	MNIST	MNIST	MNIST
LUT	1745	16,360	10,355	36,798	23,258	21,235
DSP	17	120	58	214	185	10
BRAM	10	42.5	27	123	132	48
Chip type	Xc7a35t	Zedboard	xc6slx45	Pynq-Z1	Pynq-Z1	Nexys

**Table 4:** Comparison of power consumption of CNN module design.

Platform	Power Consumption
This article	0.03 W
Document [18]	2.78 W
Document [19]	1.66 W
Document [21]	1.59 W
Document [22]	0.419 W

## Data Availability

Not applicable.

## References

[1] Rajaraman S., Candemir S., Kim I., Thoma G., Antani S. (2018). Visualization and interpretation of convolutional neural network predictions in detecting pneumonia in pediatric chest radiographs. Appl. Sci..

[2] Karpathy A., Toderici G., Shetty S., Leung T., Sukthankar R., Fei-Fei L. Large-scale video classification with convolutional neural networks. Proceedings of the IEEE Conference on Computer Vision and Pattern Recognition.

[3] Iandola F.N., Han S., Moskewicz M.W., Ashraf K., Dally W.J., Keutzer K. (2016). SqueezeNet: AlexNet-level accuracy with 50× fewer parameters and <0.5 MB model size. arXiv.

[4] Howard A.G., Zhu M., Chen B., Kalenichenko D., Wang W., Weyand T., Andreetto M., Adam H. (2017). Mobilenets: Efficient convolutional neural networks for mobile vision applications. arXiv.

[5] Howard A., Zhmoginov A., Chen L.C., Sandler M., Zhu M. (2018). Inverted Residuals and Linear Bottlenecks: Mobile Networks for Classification, Detection and Segmentation. arXiv.

[6] Zhang X., Zhou X., Lin M., Sun J. Shufflenet: An extremely efficient convolutional neural network for mobile devices. Proceedings of the IEEE Conference on Computer Vision and Pattern Recognition, Salt Lake City.

[7] Mehta S., Rastegari M., Caspi A., Shapiro L., Hajishirzi H. Espnet: Efficient spatial pyramid of dilated convolutions for semantic segmentation. Proceedings of the European Conference on Computer Vision (ECCV).

[8] Nomura O., Morie T. Projection-field-type vlsi convolutional neural networks using merged/mixed analog-digital approach. Proceedings of the International Conference on Neural Information Processing.

[9] Zhu J., Sutton P. FPGA implementations of neural networks—A survey of a decade of progress. Proceedings of the International Conference on Field Programmable Logic and Applications.

[10] Chen Y., Luo T., Liu S., Zhang S., He L., Wang J., Li L., Chen T., Xu Z., Sun N. Dadiannao: A machine-learning supercomputer. Proceedings of the 2014 47th Annual IEEE/ACM International Symposium on Microarchitecture.

[11] Yu J., Hu Y., Ning X., Qiu J., Guo K., Wang Y., Yang H. Instruction driven cross-layer CNN accelerator with winograd transformation on FPGA. Proceedings of the 2017 International Conference on Field Programmable Technology (ICFPT).

[12] Yu J., Ge G., Hu Y., Ning X., Qiu J., Guo K., Wang Y., Yang H. (2018). Instruction driven cross-layer cnn accelerator for fast detection on fpga. ACM Trans. Reconfigurable Technol. Syst. TRETS.

[13] Xiao Q., Liang Y., Lu L., Yan S., Tai Y.W. Exploring Heterogeneous Algorithms for Accelerating Deep Convolutional Neural Networks on FPGAs. Proceedings of the Design Automation Conference.

[14] Lu L., Liang Y., Xiao Q., Yan S. Evaluating fast algorithms for convolutional neural networks on FPGAs. Proceedings of the 2017 IEEE 25th Annual International Symposium on Field-Programmable Custom Computing Machines (FCCM).

[15] Zhang C., Prasanna V. Frequency domain acceleration of convolutional neural networks on CPU-FPGA shared memory system. Proceedings of the 2017 ACM/SIGDA International Symposium on Field-Programmable Gate Arrays.

[16] Rui F., Jiahe L., Zhihui X., Guangwen Y. (2015). Design of FPGA Parallel Acceleration Scheme for Convolutional Neural Networks. Comput. Eng. Appl..

[17] Gan F., Hu Z., Song C., Feng W. Energy-efficient and high-throughput FPGA-based accelerator for Convolutional Neural Networks. Proceedings of the 2016 13th IEEE International Conference on Solid-State and Integrated Circuit Technology (ICSICT).

[18] Yaozong T. (2019). Design and Implementation of FPGA-Based Convolutional Neural Network Gas Pedal. Ph.D. Thesis.

[19] Bingchen H.L.L. (2019). An FPGA implementation of a mobile convolutional neural network. Microelectron. Comput..

[20] Xiaokang B. (2019). Design and Implementation of FPGA-Based Hardware Acceleration Circuit for Convolutional Neural Network Image Classification Algorithm.

[21] Binfeng L. (2019). Design and FPGA Implementation of a Convolutional Neural Network Acceleration Circuit.

[22] Hui L. (2021). Convolutional Neural Network Research and FPGA Implementation for Handwritten Digit Recognition.

